# Advances in multiple omics of natural-killer/T cell lymphoma

**DOI:** 10.1186/s13045-018-0678-1

**Published:** 2018-12-04

**Authors:** Jie Xiong, Wei-Li Zhao

**Affiliations:** 1 State Key Laboratory of Medical Genomics, Shanghai Institute of Hematology, Shanghai Rui Jin Hospital, Shanghai Jiao Tong University School of Medicine, 197 Rui Jin Er Road, Shanghai, 200025 China; 2 Pôle de Recherches Sino-Français en Science du Vivant et Génomique, Laboratory of Molecular Pathology, Shanghai, China

**Keywords:** Natural-killer/T cell lymphomas, Genomics, Transcriptomics, Epigenomics, Metabolomics, Targeted therapy

## Abstract

Natural-killer/T cell lymphoma (NKTCL) represents the most common subtype of extranodal lymphoma with aggressive clinical behavior. Prevalent in Asians and South Americans, the pathogenesis of NKTCL remains to be fully elucidated. Using system biology techniques including genomics, transcriptomics, epigenomics, and metabolomics, novel biomarkers and therapeutic targets have been revealed in NKTCL. Whole-exome sequencing studies identify recurrent somatic gene mutations, involving RNA helicases, tumor suppressors, JAK-STAT pathway molecules, and epigenetic modifiers. Another genome-wide association study reports that single nucleotide polymorphisms mapping to the class II MHC region on chromosome 6 contribute to lymphomagenesis. Alterations of oncogenic signaling pathways janus kinase-signal transducer and activator of transcription (JAK-STAT), nuclear factor-κB (NF-κB), mitogen-activated protein kinase (MAPK), WNT, and NOTCH, as well as epigenetic dysregulation of microRNA and long non-coding RNAs, are also frequently observed in NKTCL. As for metabolomic profiling, abnormal amino acids metabolism plays an important role on disease progression of NKTCL. Of note, through targeting multiple omics aberrations, clinical outcome of NKTCL patients has been significantly improved by asparaginase-based regimens, immune checkpoints inhibitors, and histone deacetylation inhibitors. Future investigations will be emphasized on molecular classification of NKTCL using integrated analysis of system biology, so as to optimize targeted therapeutic strategies of NKTCL in the era of precision medicine.

## Background

Natural-killer/T cell lymphoma (NKTCL) is a highly aggressive subtype of non-Hodgkin’s lymphoma with malignant proliferation of CD56+/cytoCD3+ lymphocytes [1, 2]. Epstein-Barr virus (EBV) is critically involved in NKTCL and evidenced by in situ hybridization for EBV-encoded small RNA [3]. As the most common extranodal lymphoma, NKTCL occurs predominantly in nasal/paranasal area (such as the nasal cavity, nasopharynx, paranasal sinuses, tonsil, Waldeyer ring, and oropharynx), with a geographic prevalence in Asian and South American populations [2]. NK and T cells share a common bi-potential T/NK progenitor [4]. Approximately 40% of NKTCL is identified as T cell-origin, characterized by rearrangements of T cell receptor (TCR) gene and expression of TCR protein [5]. As for other cytogenetic and genetic alterations, deletion of chromosome 6q21, as well as mutations of oncogenes (*KRAS*, *NRAS*, *FAT4*, and *CTNNB*) and tumor suppressor genes (*TP53*), are frequently observed in NKTCL [6–9]. However, the driven changes of NKTCL pathogenesis and their underlying mechanisms remain to be fully elucidated.

System biology, consisting of genomics, transcriptomics, epigenomics, and metabolomics, is a group of hallmark techniques in current cancer research and provides insights into the panorama view of biological processes under malignant progression [10, 11]. These omics methods have been successfully implicated not only to elucidate pathogenesis of human diseases, but also to identify prognostic and therapeutic biomarkers [12, 13]. Here, the application of system biology on identification of multiple omics aberrations and their potential clinical rationales are reviewed in NKTCL.

### Genomic aberrations

**Fig. 1:**
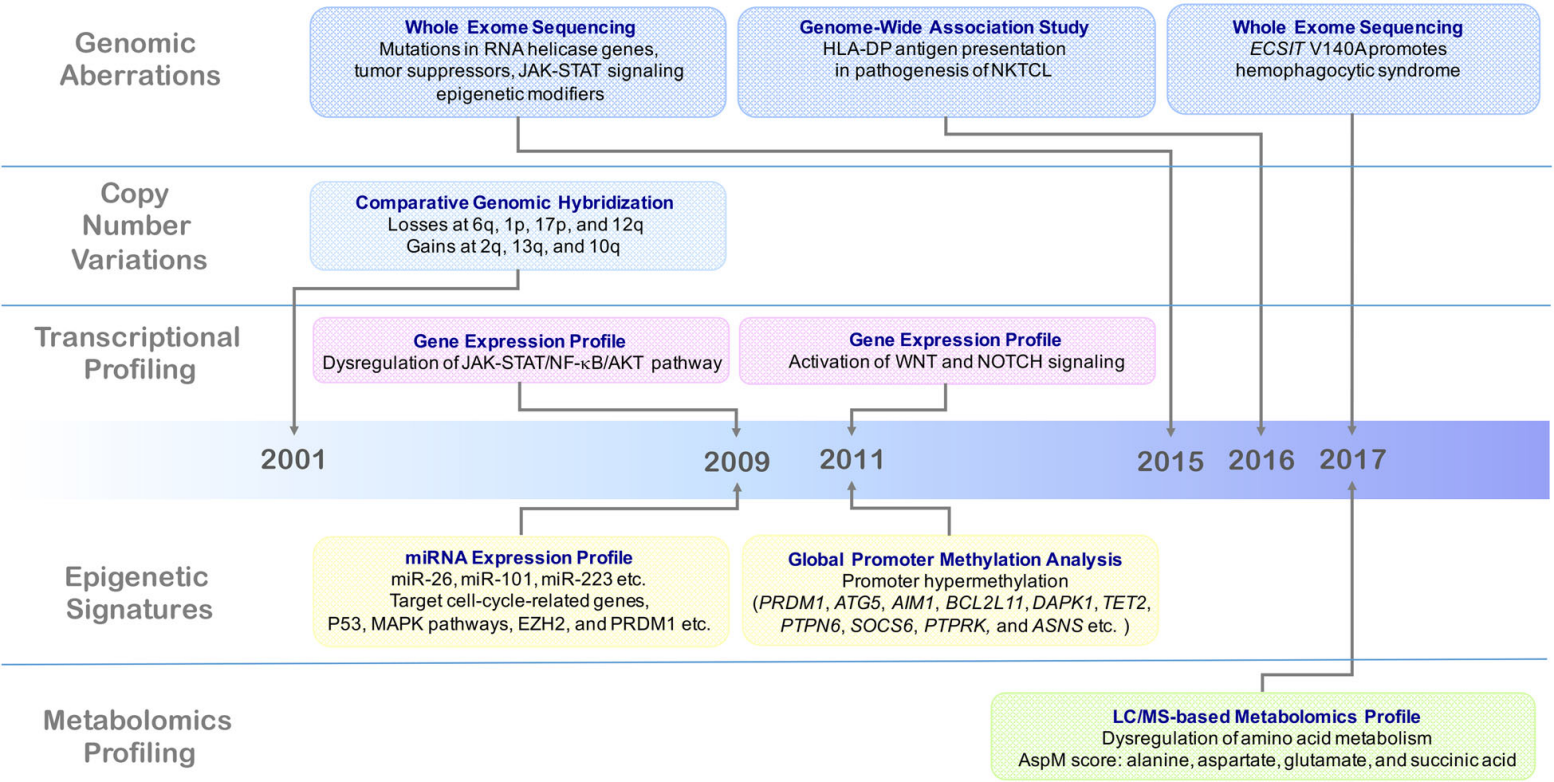
Milestones of multiple omics studies on NKTCL. This timeline describes key discoveries of genomic (whole-exome sequencing, genome-wide association study, and comparative genomic hybridization), transcriptomic (gene expression profile), epigenomic (miRNA expression profile and global promoter methylation analysis), and metabolomics (LC/MS-based metabolomics profile) studies in NKTCL

The development of multiple omics studies on NKTCL are illustrated in Fig. 1. Using whole-exome sequencing and targeted sequencing, recurrent somatic gene mutations are identified in NKTCL, mainly as RNA helicase gene *DDX3X*, tumor suppressors (*TP53*, *MGA*, and *BCOR*), janus kinase-signal transducer and activator of transcription (JAK-STAT) pathway molecules (*JAK3*, *STAT3*, and *STAT5B*), and epigenetic modifiers (*MLL2*, *ARID1A*, *EP300*, and *ASXL3*) [9, 14]. Of note, *DDX3X* mutants exhibit decreased RNA-unwinding activity, loss of suppressive effects on cell-cycle progression in NK cells, as well as transcriptional activation of nuclear factor-κB (NF-κB) and mitogen-activated protein kinase (MAPK) pathways. Patients with mutations in *DDX3X* and *TP53* have a poor response to anthracycline-based chemotherapy [14]. Functioned as a tumor suppressor, *MGA* gene inhibits MYC-dependent cell growth and malignant transformation through binding with MAX [15]. Somatic loss-of-function mutations of *MGA* have been observed in solid tumors and may lead to tumor development [16]. *BCOR* is also likely to play an important role as a tumor suppressor gene [17]. However, the pathogenic mechanism of *MGA* and *BCOR* has not yet been revealed in NKTCL. *JAK3*-activating mutations are involved in cytokine-independent JAK-STAT signaling pathway activation to enhance NKTCL cell proliferation [18, 19]. *STAT3* mutations are associated with STAT signaling pathway activation, and confer high programed death ligand 1 (PD-L1) expression, which may promote tumor immune evasion [20, 21]. Mutations in genes related to epigenetic modification of NKTCL include histone methylation (*KMT2D*), histone acetylation (*EP300*), histone deubiquitination (*ASXL3*), and chromatin remodeling (*ARID1A*) [22]. A case with extranodal EBV-negative NKTCL is reported to harbor *KDM6A* mutation, which is located on Xp11.2 and acts as an enzyme specifically demethylating H3K27 [23].

Through genome-wide association study, genetic variants affecting individual risk of NKTCL has been investigated, showing that single nucleotide polymorphisms mapping to the class II MHC region on chromosome 6, with rs9277378 located in *HLA-DPB1* is the strongest contributor to lymphomagenesis (odds ratio 2.65) [24]. More recently, a hotspot mutation of *ECSIT*-V140A has also been identified in NKTCL patients with lymphoma-associated hemophagocytic syndrome and poor prognosis [25].

### Copy number variations (CNVs)

Accumulation of genomic imbalances is implicated in hematological malignancies inducing the activation of oncogenes or inactivation of tumor suppressor genes. As revealed by comparative genomic hybridization, 6q21 is frequently deleted in NKTCL, leading to the loss of tumor suppressor genes located in this region, including *PRDM1*, *ATG5*, *AIM1*, *FOXO3*, and *HACE1* [26, 27]. *PRDM1* is required for NK-cell maturation and proliferation [28]. Mutation or methylation in *PRDM1*, *ATG5*, and *AIM1* have been reported in NKTCL cell lines [29], while another study indicates that *HACE1* is not directly related to NKTCL pathophysiology [30].

Besides, recurrent CNVs are observed in other regions of chromosomes, comprising of chromosomal losses (on 1p, 17p, and 12q) and gains (on 2q, 13q, and 10q) [31]. Involved chromosomal fragments may include candidate genes related to malignant transformation and invasion (*S100A16*, *LAMB1*, *LAMC1*, *COL1A2*, and *CTSB*), cell-cycle progression (*CCND3*), JAK-STAT (*AKT3*, *IL6R*, and *CCL2*), and NF-κB (*PRKCQ* and *TNFRSF21*) signaling pathways [32]. More recently, other molecular clusters have been proposed, such as loss of 14q11.2 (TCRA loci), gain of 1q32.1-q32.3, and loss of Xp22.33 [33].

### Transcriptional profiling

Based on gene expression profiling, integrations of JAK-STAT, NF-κB, and AKT signaling pathways contribute to genotoxic stress, angiogenesis, immunosuppression, and disease progression of NKTCL, as compared to normal NK cells [32, 34]. Activation of WNT and NOTCH signaling pathways are also enriched in NK-cell malignancies [35]. In according with CNV findings, downregulation of tumor suppressor genes in 6q21 (*PRDM1*, *ATG5*, *AIM1*) are confirmed by microarray analysis [27, 32]. As for individual genes, it is noteworthy that MYC induces upregulation of *EZH2* and *RUNX3*, both of which exert cascade effect of transcriptional activation during lymphomagenesis [36, 37]. Using RNA sequencing technology, overexpression of *KIR2DL4* is reported in malignant NK cells [38]. *KIR2DL4* mediates NK-cell activation via inducing proliferation and survival pathways such as NF-κB and AKT, which may contribute to NKTCL pathogenesis [38].

### Epigenetic signatures

In addition to mutations in epigenetic modifiers, differential expression of miRNAs plays a pathogenic role in NKTCL, through targeting cell-cycle-related genes, P53 and MAPK signaling pathways [39, 40]. Loss of miR-26 and miR-101 contribute to the overexpression of *EZH2*, while upregulation of miR-223 downregulates *PRDM1* [36, 41]. EBV-encoded miRNAs have also been detected, including miRs-BART 1 to 22 of BamHI-A region rightward transcript (BART) family, as well as miRs-BHRF1-1, miRs-BHRF1-2, and miRs-BHRF1-3 of the BamHI fragment H rightward open reading frame 1 (BHRF1) family [42, 43]. Viral miRNAs are relatively less present in NKTCL than in nasopharyngeal carcinoma (2.3% of the total miRNA reads vs 5–19% in nasopharyngeal carcinoma) with unknown function [42, 44]. Meanwhile, NKTCL-associated dysregulated long non-coding RNAs have been identified, such as *SNHG5*, *ZFAS1*, and *MIR155HG* [45]. Among them, upregulation of *ZFAS1* is implicated in stabilization of TP53, alterations of apoptosis and cell cycle, and activation of NF-κB signaling, while *MIR155HG* is downregulated by PRDM1 in NKTCL [45].

Promoter region hypermethylation has been investigated by global methylation assays, locus-specific validation of methylation, and methylation-specific polymerase chain reaction, demonstrating increased methylation and decreased gene expression with pathological and clinical significance, including *PRDM1*, *ATG5*, *AIM1*, *BCL2L11*, *DAPK1*, *TET2*, *PTPN6*, *SOCS6*, *PTPRK*, and *ASNS* [27, 46, 47]. Functionally, inactivation of *TET2* may contribute to hypermethylation of global promoters in NKTCL [46]. *PTPN6*, *SOCS6*, and *PTPRK* negatively regulate JAK-STAT, suggestive an alternative mechanism responsible for activation of JAK-STAT signaling pathway [46–49].

### Metabolomics profiling

Serum metabolomic profile of NKTCL patients is distinct from that of healthy volunteers [50]. Briefly, 115 significantly altered serum metabolites are identified, predominantly involving in pathways of amino acid metabolism [50]. As depicted by alanine, aspartate, and glutamate metabolism pathway in KEGG (Kyoto Encyclopedia of Genes and Genomes), nine of them are asparaginase-associated metabolites (alanine, aspartic acid, malic acid, ornithine, glutamate, glutamine, histidine, pantothenic acid, and succinic acid) and differently expressed in patients with good response to asparaginase, suggesting the reliance of malignant NK cells on extracellular amino acids. Based on serum metabolomics, our group has established a prognostic asparaginase-associated metabolic (AspM) score, including alanine, aspartate, glutamate, and succinic acid [50]. As a prognostic score independent of International prognostic index, as well as prognostic index of natural-killer lymphoma (PINK) or PINK in combination with peripheral blood EBV DNA, AspM score is easily attainable from peripheral blood and efficiently predicts response to asparaginase-based regimens [50].

### Therapeutic strategies targeting multiple omics alterations

**Fig. 2:**
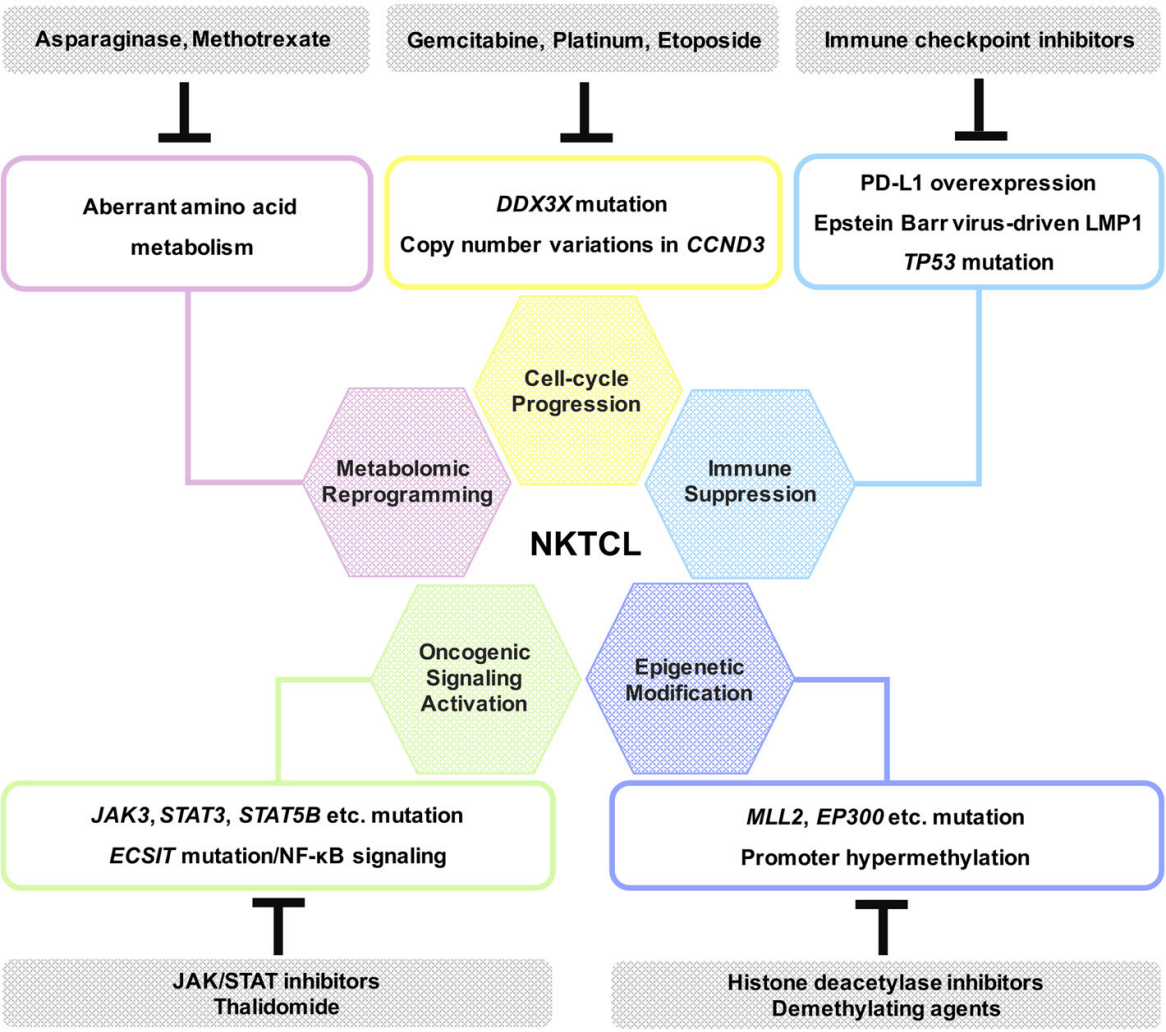
Schematic description of NKTCL pathogenesis and targeted therapeutic strategies. This illustration encompasses six hallmark mechanisms involved in NKTCL pathogenesis, which are closely related to targeted therapeutic strategies

Schematic description of NKTCL pathogenesis and targeted therapeutic strategies are shown in Fig. 2. With the understanding of multiple omics alterations, clinical outcome of NKTCL has been significantly improved by new therapeutic strategies.

Different from metabolomic fingerprints of T and B cell lymphoma, NKTCL is characterized by dysregulated amino acid metabolism, mainly as alanine, aspartate, and glutamate metabolism [50]. Asparaginase and methotrexate are the most commonly used anti-metabolite agents, functioning through hydrolyzing extracellular asparagine or targeting folate, pyrimidine, and purine metabolism, respectively [50, 51]. For localized NKTCL, methotrexate, etoposide, dexamethasone, and peg-asparaginase (MESA) sandwiched with radiotherapy achieved complete remission (CR) rate of 89.5% in 38 patients. The 2-year progression-free survival (PFS) and overall survival (OS) rate are 89.1% and 92.0% [50]. These data highlight the role of targeting metabolic vulnerability in NKTCL.

Increased expression of cell cycle-related genes has been reported in NKTCL [39]. Platinum, gemcitabine, and etoposide are cell cycle-specific DNA damaging agents [52–54], which are prevalently used in NKTCL chemotherapy. For advanced or relapsed/refractory NKTCL, CR rate of P-GEMOX (peg-asparaginase, gemcitabine, and oxaliplatin) is 51.4% of 35 patients, with 2-year PFS and OS rate of 38.6% and 64.7% [55]. In a randomized controlled, multicenter, and open-label clinical trial, DDGP (dexamethasone, cisplatin, gemcitabine, and peg-asparaginase) results in a CR rate of 71%, as well as significant improvement in 2-year PFS and OS rate to 86% and 74% [56]. Therefore, inhibition of cell-cycle progression is another key target in treating NKTCL [57].

Programmed death ligand 1 (PD-L1) is frequently upregulated in NKTCL [33]. Moreover, *TP53* mutation, activation of STAT3 signaling pathway, and EBV-driven latent membrane protein-1 are all related to PD-L1 overexpression [20, 58, 59]. Clinically, patients with NKTCL relapsed or refractory from l-asparaginase-based regimens and allogeneic hematopoietic stem-cell transplantation respond well to the anti-programmed death-1 (PD-1) antibody pembrolizumab, with overall response rate (ORR) as 100% [60]. Favorable responses to pembrolizumab are also observed in another independent study with ORR as 57% (4 out of 7 relapsed/refractory NKTCL) [61], indicating that PD-1 blockade is an important immunotherapy for NKTCL resistant to anti-metabolic and cytotoxic agents.

Histone deacetylase inhibitors serve as promising epigenetic agents, and phase II trials have been carried out in T cell lymphoma (including NKTCL), showing that 1 out of 2 enrolled NKTCL cases responds to Belinostat, while 3 out of 16 cases respond to Chidamide [62, 63]. Since promoter region hypermethylation is present in NKTCL, in vitro studies indicate that reversal of methylation by decitabine induces expression of key candidate genes involved in tumor suppressor (*PRDM1*), pro-apoptosis (*BIM* and *SAPK*), JAK-STAT pathway (*SOCS6*, *ZFHX3*, and *PTPN6*), and cell growth inhibition (*CD300A*) etc., leading to increased NK-cell death [27, 46].

*ECSIT-V140A* is associated with activation of NF-κB pathway, transcription, and secretion of pro-inflammatory cytokines. The immunomodulatory agent thalidomide prevents NF-κB from binding to the promoters of its target genes (including TNF and IFNG), and combined treatment of thalidomide and dexamethasone extends disease-free survival of two NKTCL patients with hemophagocytic syndrome who express *ECSIT-V140A* for longer than 3 years [25]. Lenalidomide has also successfully been used in a patient with relapsed NKTCL after autologous hematopoietic stem-cell transplantation [64].

Novel bio-agents are currently under pre-clinical studies. High-throughput drug sensitivity and resistance testing identify JAK inhibitor ruxolitinib to be highly effective across NKTCL cell lines [65]. Therapeutic effect of a novel selective JAK3 inhibitor PRN371 has been recently confirmed in xenograft model harboring *JAK3* activating mutation [66]. As mechanism of action, JAK3 inhibitors inhibit NKTCL cell growth in an EZH2 phosphorylation-dependent manner, which functions as a transcriptional activator of NKTCL. STAT3 inhibitor tofacitinib is active against *STAT3*-mutant NKTCL cell lines [18], while JAK1/2 inhibitor partially against *STAT3* and *STAT5B* mutations [21]. STAT3 activation confers PD-L1 overexpression, which can be downregulated by STAT3 inhibitors, alone or combined with PD-1/PD-L1 antibodies [20]. Combined treatment of LEE011 and ruxolitinib synergistically inhibit NKTCL cell growth, suggesting that targeting of both CDK4/6 and JAK1/2 are promising treatment alternatives for NKTCL [67].

### Perspectives

Multiple omics analysis reveals genetic, epigenetic, transcriptomic and metabolic aberrations, which are not only associated with disease progression, but also response to clinical management. In the future, integration of system biology techniques should be further carried out to classify disease into subtypes of distinct molecular fingerprints, paving way for the implication of mechanism-based targeted therapy in NKTCL.

## Abbreviations

AspM: Asparaginase-associated metabolic score
BART: BamHI-A region rightward transcript
BHRF1: BamHI fragment H rightward open reading frame 1
CNV: Copy number variation
CR: Complete remission
EBV: Epstein-Barr virus
JAK-STAT: Janus kinase-signal transducer and activator of transcription
MAPK: Mitogen-activated protein kinase
NF-κB: Nuclear factor-κB
NKTCL: Natural-killer/T cell lymphoma
ORR: Overall response rate
OS: Overall survival
PD-1: Programmed death 1
PD-L1: Programed death ligand 1
PFS: Profression-free survival
PINK: Prognostic index of natural-killer lymphoma
TCR: T cell receptor
